# Identification of Key Histone Modifications and Their Regulatory Regions on Gene Expression Level Changes in Chronic Myelogenous Leukemia

**DOI:** 10.3389/fcell.2020.621578

**Published:** 2021-01-12

**Authors:** Lu-Qiang Zhang, Guo-Liang Fan, Jun-Jie Liu, Li Liu, Qian-Zhong Li, Hao Lin

**Affiliations:** ^1^Laboratory of Theoretical Biophysics, School of Physical Science and Technology, Inner Mongolia University, Hohhot, China; ^2^The Research Center for Laboratory Animal Science, College of Life Sciences, Inner Mongolia University, Hohhot, China; ^3^Key Laboratory for Neuro-Information of Ministry of Education, Center for Informational Biology, School of Life Sciences and Technology, University of Electronic Science and Technology of China, Chengdu, China

**Keywords:** chronic myelogenous leukemia, histone modification, gene expression level changes, H3K79me2, H3K36me3

## Abstract

Chronic myelogenous leukemia (CML) is a type of cancer with a series of characteristics that make it particularly suitable for observations on leukemogenesis. Research have exhibited that the occurrence and progression of CML are associated with the dynamic alterations of histone modification (HM) patterns. In this study, we analyze the distribution patterns of 11 HM signals and calculate the signal changes of these HMs in CML cell lines as compared with that in normal cell lines. Meanwhile, the impacts of HM signal changes on expression level changes of CML-related genes are investigated. Based on the alterations of HM signals between CML and normal cell lines, the up- and down-regulated genes are predicted by the random forest algorithm to identify the key HMs and their regulatory regions. Research show that H3K79me2, H3K36me3, and H3K27ac are key HMs to expression level changes of CML-related genes in leukemogenesis. Especially H3K79me2 and H3K36me3 perform their important functions in all 100 bins studied. Our research reveals that H3K79me2 and H3K36me3 may be the core HMs for the clinical treatment of CML.

## Introduction

Chronic myelogenous leukemia (CML) is a malignant hematopoietic stem cell disease of the bone marrow and owns a series of characteristics that make it particularly suitable for observations on leukemogenesis (Liu et al., 2015; Radivoyevitch et al., 2020; Wu et al., 2020). It is characterized by the (9:22) translocation and resultant production of the constitutively activated BCR-ABL tyrosine kinase (Vos et al., 2016; Wang et al., 2016; American Cancer Society, 2019). As a subtype of leukemia, the annual incidence of CML is one to two cases per 100,000 adults, accounting for 15–25% of newly diagnosed adult leukemia cases and 14% of overall leukemia (American Cancer Society, 2019). In the past two decades, due to the discovery of targeted drugs, such as imatinib mesylate (O’Brien et al., 2003; Rousselot et al., 2007), the 5 years survival rate of CML has increased from 31% for patients diagnosed in the early 1990s to 68% for those diagnosed from 2007 to 2013 (American Cancer Society, 2019). Though the application of targeted drugs is expected to overcome CML, the persistence of leukemia stem cells indicates that additional strategies for treating CML need to be researched.

Studies have revealed that the pathogenesis of CML is associated with the activation of oncogenes and inactivation of tumor suppressor genes. The loss-of-function mutations of these CML-related genes are linked to the dynamic alterations of histone modifications (HMs) (Zhang and Li, 2017, 2020; Zhang et al., 2017, 2018). As an integral part of HMs, histone acetylation and methylation are the most investigated modifications that are reversible, and the aberrations of HMs and the mutations of their modulators are associated with leukemogenesis (Zhang et al., 2004; Funata et al., 2017). The loss of histone H3 and H4 acetylation is attributed to the imbalanced recruitment of histone deacetylases and results in transcription repression of tumor suppressors in leukemia (Esteller, 2007). For example, promoter histone hypoacetylation leads to PDH1 silencing (Ma et al., 2010) and decreases the mRNA and protein level of BCR-ABL in CML and LAMA-84 cells (Nimmanapalli et al., 2003), while hyperacetylation induces the expression of p21 and/or p27 (Polakova et al., 2013).

As reported in a series of recent publications (Bu et al., 2018; Dhall et al., 2019), the traditional methods used to identify core HMs and their regulatory regions are based on immunoassay techniques. Although these methods are sensitive and precise, they require expensive instrumentation, time-consuming processes, well-trained personnel, and site-specific antibodies. In this study, we first analyze the distribution differences and employ statistical analyses for 11 HM signals in CML cell lines as compared with that in normal cell lines. On this basis, we explore the effects of HM signal changes in various genomic regions on gene expression changes. Finally, based on the signal changes of HMs in leukemogenesis, random forest (RF) algorithm, and subset construction, the key HMs and genomic regions are identified. Our study provides a better understanding of the impacts of HMs on gene expression level changes in CML and theoretical guidance for the clinical research of CML.

## Materials and Methods

### Data

The human genome location information (hg19) is downloaded from the UCSC database. Genes encoding mature messenger RNA are chosen out. To avoid the possibility that some genes may be the alternative transcripts of the same gene, only one gene with the same name is kept. The genome-wide profiles of 11 HMs and polyA plus RNA-seq data in GM12878 (B-lymphoblastoid cell, normal) and K562 (CML cell, cancer) are deposited in the ENCODE database. The corresponding accession numbers are displayed in Supplementary Table S1. For visualization, the raw bam-format data is converted to bed format by using the BEDtools software (Quinlan and Hall, 2010).

### Formulation of the Histone Modification Signal Levels

For the *i*-th gene, the reads number of the *k*-th HM in DNA regions flanking the transcription start site (TSS) (−5 to 5 kb) is normalized by Eq. (1).

(1) $$H_{i}^{k}=\left(h_{i}^{k}\times 10^{9}\right)/\left(h_{k}\times L\right)$$

in which $H_{i}^{k}$ represents the normalized signal level, and $h_{i}^{k}$ is the reads number of the *k*-th HM mapped into the DNA regions flanking the TSS of the *i*-th gene. *h*_*k*_ denotes the *k*-th HM sequencing depth, *L* is the length of the DNA regions flanking the TSS. 10^9^ is used to keep the consistent magnitude with RPKM (in the process of calculating RPKM, the unit of the exon length is kilobase and the counting unit of mapped reads is million).

To further investigate the signal distributions and roles of 11 HMs in CML, we divide the DNA regions flanking the TSS into 100 bins, each of 100 bp in size. Then, the signals of HMs are normalized by using the following Eq. (2),

(2) $$H_{i\,j}^{k}=\left(h_{i,j}^{k}\times 10^{9}\right)/\left(h_{k}\times L_{j}\right)$$

Where $H_{i\,j}^{k}$ represents the signal level of the *k*-th HM in the *j*-th bin of the *i*-th gene. $h_{i,j}^{k}$ is the total reads that the *k*-th HM locates in the *j*-th bin of the *i*-th gene, and *L*_*j*_ is the length of the *j*-th bin. The HM signal levels in DNA regions flanking the TSS or in each bin are averaged for biological replicates.

### Correlation Analysis of Histone Modification Signals and Gene Expression Level Changes

For analyzing the relations between HM signals in different bins and gene expression level changes, we first use the “DEGSeq” R package to calculate the RPKM value of each RefSeq gene in normal and CML cell lines through polyA plus RNA-seq data. The RPKM value (reads per kilobase of exon model per million mapped reads) describes gene expression level (Mortazavi et al., 2008). Next, the differential expression genes (DEGs) between normal and CML cell lines are identified by the “DESeq2” R package. A total of 2,267 genes with adjusted *p* < 0.01 and log_2_(FC) > 1 are defined as up-regulated DEGs (up-DEGs), and 2,567 genes with adjusted *p* < 0.01 and log_2_(FC) < −1 are judged as down-regulated DEGs (down-DEGs). Then, for the up-/down-regulated DEGs, according to the ratios of the HM signals/gene expression levels in CML cells to that in normal cells, we implement Spearman correlation analysis of HM signal changes in each bin and gene expression level changes. Similarly, the relations among any pair of HMs in DNA regions flanking the TSS were also performed by Spearman correlation analysis.

### The Prediction of Up-DEGs and Down-DEGs in Chronic Myelogenous Leukemia

For exploring the impacts of various HMs on the expression level changes of leukemia-related genes, we employ the signal changes of HMs to predict the up- and down-DEGs in CML through the random forest algorithm. The random forest algorithm is built by bootstrap samples and designed to accommodate non-linearities between the prediction variables and response, which can robustly avoid the over-fitting phenomenon (Mehrmohamadi et al., 2016). The studied DEGs are randomly selected with two-thirds as the training set and the rest as the testing set. The random forest model is built in the training set and subsequently applied to the testing set to predict the up- and down-DEGs. To test the stability of these predictions, the above-mentioned procedures are repeated 10 times. Based on the average sensitivity (*S*_n_) and specificity (*S*_p_), we calculate the area under the receiver operating characteristic curve (AUC). The AUC is finally adopted to measure the impacts of HM signal changes on the expression level changes of leukemia-related genes.

(3) $$\begin{array}{c} S_{n}=1-N_{D}^{U}\,/\,N^{U}\\ S_{p}=1-N_{U}^{D}\,/\,N^{D} \end{array}$$

Where *N*^*U*^ and *N*^*D*^ are the number of up- and down-DEGs in the testing dataset, respectively. $N_{D}^{U}$ is the number of up-DEGs that are incorrectly recognized as down-DEGs, and $N_{U}^{D}$ is the number of down-DEGs that are incorrectly recognized as up-DEGs.

### Statistical Analysis

The interaction network among HMs is built by Cytoscape software (Shannon et al., 2003). Student’s *t*-test is utilized, and *P*-values less than 0.01 are considered to be statistically significant. To measure the contributions of each of the 11 HMs in the same bin to the expression level change of CML-related genes, the percent increase in the mean squared error (IncMSE) is calculated. Due to the non-sense of “IncMSE” values when they are considered outside of the current bins, we rank the information parameters by converting their “IncMSE” values to orders. Information parameters with lower rank values indicate higher contributions. Cluster analysis is employed by Euclidean distance (Liberti et al., 2014). The R/Bioconductor software packages and “Origin_9.1” are used for data statistical analysis and result visualization.

## Results

### Histone Modification Signal Levels Vary Obviously in Chronic Myelogenous Leukemia

**FIGURE 1:**
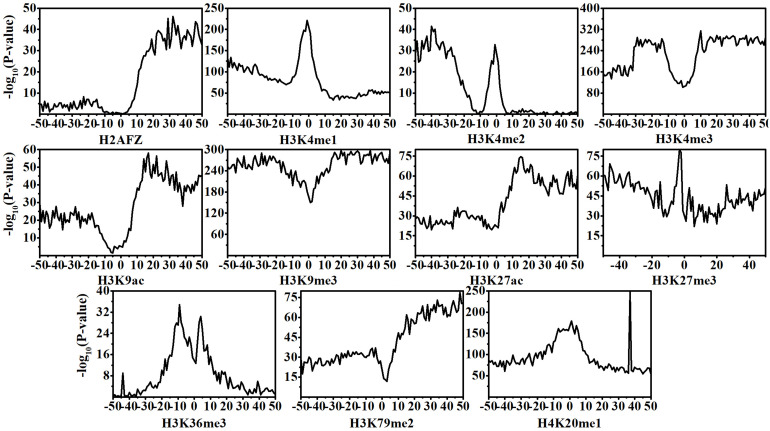
Statistical differences of histone modification signal levels between normal and chronic myelogenous leukemia cells across the 100 bins within the DNA regions flanking transcription start site (TSS). Position 0 represents the TSS.

Recent investigators have revealed that the pathogenesis of CML is closely related to the dynamic changes of HMs (Zhang and Li, 2017, 2020; Zhang et al., 2017, 2018). In this research, according to Eq. (2) and Student’s *t*-test, we calculate and investigate the distributions and statistical difference (*p*-value) of HM signals across the 100 bins within normal and CML cell lines. The results are shown in Figure 1. Among the 11 HMs, except H2AFZ and H3K4me2, the rest of the HMs display remarkably dynamic changes in CML cells as compared with that in normal cells. The changes of H2AFZ signals mostly appear in the downstream regions of TSS, and H3K4me2 signal changes are significantly concentrated in the upstream regions of TSS.

### Correlation Analysis of Histone Modification in Various Regions and Gene Expression Level Changes in Chronic Myelogenous Leukemia

**FIGURE 2:**
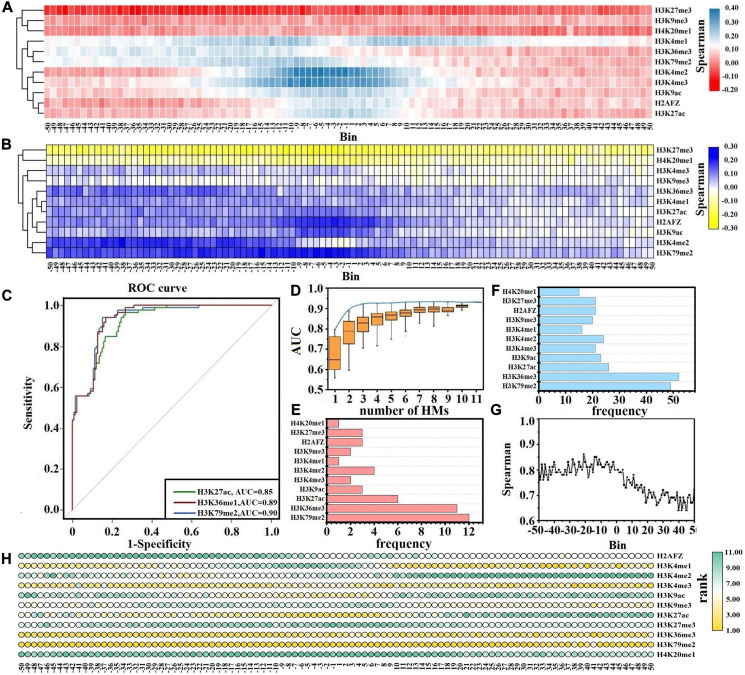
Correlation analysis between histone modification (HM) signal changes and gene expression changes. Spearman correlation analysis of HM signal changes and gene expression changes for down-regulated differentially expressed genes (DEGs) **(A)** and up-regulated DEGs **(B)**. **(C)** Receiver operating characteristic (ROC) curves for H3K79me2, H3K36me3, and H3K27ac. **(D)** The distributions of area under the ROC curve (AUCs) for all possible combinations of HMs. The blue curve describes the best prediction accuracy of the one-HM until 11-HM models. The frequency of each HM in the studied three-HM model **(E)** and four-HM models **(F)**. **(G)** AUCs in the 100 bins flanking the transcription start site. **(H)** The rank for the predictive ability of each HM across the 100 bins. A HM with a lower rank (higher IncMSE) value indicates a higher predictive ability for gene expression changes.

Previous studies and our findings have reported that the changes in HM signals play key roles in gene expression changes (Zhang et al., 2011, 2018; Liu et al., 2013; Zhang and Li, 2017, 2020). To investigate the impacts of each HM in different bins on gene expression changes, we first identify the DEGs between normal and CML cells. Next, for the up- and down-DEGs, we calculate the ratios of HM signals/gene expression levels in CML cells to that in normal cells, respectively, and then the relations between HM signal changes and gene expression changes are implemented through Spearman correlation analysis. The results are shown in Figures 2A,B and Supplementary Figure S1.

For the down-DEGs, except H3K27me3, H3K4me3, and H4K20me1, the signals of other HMs reduce significantly in most bins within CML cells (Supplementary Figure S1A). The signals of H3K27me3 increase in all 100 bins within CML cells, and the ratio of the H3K27me3 signals in CML cells to that in normal cells reaches 1.7 in the −31st bin. The signals of H3K4me3 rise from the −15th to the 50th bins, and the maximum ratio is 1.9 in the 5th bin. The H4K20me1 signals are slightly enhanced from the −19th to the 50th bins. Cluster analysis exhibits that the impacts of HM signal changes on the expression level changes of down-DEGs are divided into two categories. The first category includes H3K27me3, H3K9me3, and H4K20me1, which are repressive epigenetic markers (Segal et al., 2018), while other HMs that promote gene expression are classified into the second category (Bernt et al., 2011). Our study shows that the increased signals of HMs in category 1 and the decreased signals of HMs within category 2 together lead to the down-regulation of gene expression (Figure 2A).

For the up-DEGs, except H3K27me3, the signals of other HMs are increased across all bins within CML cell lines (Supplementary Figure S1B). For example, the maximum ratio for H3K4me3 is 4.9 in the −9th bin, and the maximum ratio for H3K79me2 reaches 3.4 in the −27th bin. The signals of H3K27me3 slightly decrease in all 100 bins within CML cells. Cluster analysis displays that the influences of HM signal changes on the expression changes of up-DEGs are also divided into two categories. The reduced signals of HMs (especially H3K27me3) in category 1 and the increased signals of HMs in category 2 together induce the up-regulation of gene expression levels, and those HMs in category 2 have stronger inducibility within the upstream regions of TSS (Figure 2B).

### H3K79me2 and H3K36me3 Exert Their Important Regulatory Functions on Gene Expression Level Changes in All 100 Genomic Regions

Although HMs within two categories cooperatively regulate gene expression, their functions are not identical (Budden et al., 2014, 2015). For further exploring which HMs contribute more to the expression level changes of leukemia-related genes, based on the ratios of HM signals in CML cells to that in normal cells across the 100 bins, we predict the up- and down-DEGs by RF model. The AUC is used to measure the prediction abilities.

Of the 11 MHs, H3K79me2, H3K36me3, and H3K27ac achieve better prediction results, and the AUCs are greater than 0.85 (Figure 2C). Therefore, H3K79me2, H3K36me3, and H3K27ac may be the more crucial HMs in inducing the expression changes of leukemia-related genes. Across the 100 bins, H3K79me2 signals in the −13th, −9th, −7th, −18th, and −8th are relatively important for the expression regulation of leukemia-related genes; H3K36me3 signals in the −37th, −31st, −41st, −40th, and −42nd are more crucial for the expression changes of leukemia-related genes; H3K27ac signals in the −5th, −6th, −2nd, −3rd, and −8th play key roles in predicting the up- and down-DEGs in CML (Supplementary Table S2).

To validate this finding, the ratios of HM signals in CML cells to that in normal cells within the DNA regions flanking the TSS are calculated (see Eq. 1) and regarded as the input parameters to predict the up- and down-DEGs. A total of 2,047 RF models are built based on all possible combinations of HMs by choosing *n* out of the 11 HMs (*n* = 1, 2, …, 11). The predicted results of all combinations and the best prediction accuracy of the one-, two-, and until 11-HM models are displayed in Figure 2D. As shown, although models with more HMs are generally more predictive, the predictive powers will reach the summit when the models are with three types of HM. The best three-HM model includes H3K4me2, H3K36me3, and H3K79me2, and the prediction accuracy is AUC = 0.92. Though it is important to identify the best combination of HMs, we also need to consider the presence of HM which can effectively increase the predictive accuracy. We thus focus on the combinational models of three types of HMs with AUCs reaching at least 95% of the AUC of the 11-HM model. By counting the frequency of each HM in these combination models, we find that H3K79me2, H3K36me3, and H3K27ac appear more frequently (Figure 2E). The same analysis for four-HM models is supplemented, and analogous consequences are found (Figure 2F).

Besides this, we further analyze which genomic regions and which HMs in these genomic regions contribute more to the expression level changes of leukemia-related genes. For the 11 HMs in the same bin, we take the ratios of each HM signal in CML cells to that in normal cells and select them as the inputs of the RF model to predict the up- and down-DEGs. The predicted results (AUCs) are shown in Figure 2G. Within the upstream 5 kb regions of TSS, the AUCs change slightly, while in the downstream regions of TSS the AUCs change dramatically and the further away from the TSS, the worse the predictive results are. The best predictive result appears in the −21st bin. These results show that the upstream regions of TSS (especially the −21st bin) may contribute more to the regulation of gene expressions. Meanwhile, by calculating the IncMSE values for each of the 11 HMs in the same bin, we measure their contributions to the prediction of up-DEGs and down-DEGs (see Figure 2H and Table 1). It is noteworthy that H3K79me2 plays the most important role in almost all 100 bins, followed by H3K36me3. H3K27ac is relatively important for the regulation of gene expressions from the −20th to the 10th bins. H3K4me1 exerts its regulatory function from the 10th to the 50th bins. H3K4me3 has a crucial impact on gene expression changes in the −50th to −10th bins and the 20th–50th bins.

**TABLE 1:** The most important histone modifications in each of the 100 bins.

Bin	Imp_HM	Bin	Imp_HM	Bin	Imp_HM	Bin	Imp_HM
−50	H3K79me2	−25	H3K79me2	1	H3K79me2	26	H3K79me2
−49	H3K79me2	−24	H3K79me2	2	H3K79me2	27	H3K79me2
−48	H3K79me2	−23	H3K79me2	3	H3K79me2	28	H3K79me2
−47	H3K79me2	−22	H3K79me2	4	H3K79me2	29	H3K79me2
−46	H3K79me2	−21	H3K79me2	5	H3K79me2	30	H3K79me2
−45	H3K79me2	−20	H3K79me2	6	H3K79me2	31	H3K79me2
−44	H3K79me2	−19	H3K79me2	7	H3K79me2	32	H3K36me3
−43	H3K79me2	−18	H3K79me2	8	H3K79me2	33	H3K4me1
−42	H3K79me2	−17	H3K79me2	9	H3K79me2	34	H3K4me1
−41	H3K79me2	−16	H3K79me2	10	H3K79me2	35	H3K4me1
−40	H3K79me2	−15	H3K79me2	11	H3K79me2	36	H3K4me1
−39	H3K79me2	−14	H3K79me2	12	H3K79me2	37	H3K79me2
−38	H3K79me2	−13	H3K79me2	13	H3K79me2	38	H3K4me3
−37	H3K79me2	−12	H3K79me2	14	H3K79me2	39	H3K79me2
−36	H3K79me2	−11	H3K79me2	15	H3K79me2	40	H3K4me1
−35	H3K79me2	−10	H3K79me2	16	H3K79me2	41	H3K36me3
−34	H3K79me2	−9	H3K79me2	17	H3K79me2	42	H3K79me2
−33	H3K79me2	−8	H3K79me2	18	H3K79me2	43	H3K79me2
−32	H3K79me2	−7	H3K79me2	19	H3K79me2	44	H3K79me2
−31	H3K79me2	−6	H3K79me2	20	H3K79me2	45	H3K4me1
−30	H3K79me2	−5	H3K79me2	21	H3K79me2	46	H3K79me2
−29	H3K79me2	−4	H3K79me2	22	H3K79me2	47	H3K79me2
−28	H3K79me2	−3	H3K79me2	23	H3K79me2	48	H3K79me2
−27	H3K79me2	−2	H3K79me2	24	H3K79me2	49	H3K79me2
−26	H3K79me2	−1	H3K79me2	25	H3K79me2	50	H3K4me3

## Discussion

Understanding the roles and influences of HM signal changes on the expression level changes of leukemia-related genes can help to reveal new tumorigenesis mechanisms and therapeutic strategies. In this study, by analyzing the alterations of HM signals and their impacts on gene expression changes, we notice that H3K79me2, H3K36me3, and H3K27ac have crucial effects on gene expression changes. The signals of these three HMs are significantly increased or decreased in 100 bins flanking the TSS for the up- or down-DEGs within CML cells, and the signal changes of these three HMs are positively correlated with the expression changes of leukemia-related genes, especially for H3K79me2 and H3K36me3.

As important HMs, H3K79me2 is related to DNA replication initiation (Fu et al., 2013), maintaining enhancer–promoter interactions (Godfrey et al., 2019), transcriptional regulation, cell cycle regulation, and DNA damage response (Anh Tram and Zhang, 2011). H3K36me3 plays its important functions in alternative splicing (Luco et al., 2010), DNA mismatch repair (Li et al., 2013), chromatin remodeling (Pfister et al., 2014), transcription elongation (Carvalho et al., 2013; Wen et al., 2014), and DNA double-strand break repair (Carvalho et al., 2014). These functions are related to chromatin readers with proline*–*tryptophan*–*tryptophan*–*proline domains, which interact with methylated lysine residues (Vermeulen et al., 2010), and the Setd2-mediated pattern changes of H3K36me3 and H3K79me2 are associated with transcriptional deregulation of a novel set of genes including ASXL1 and ERG (Bu et al., 2018). Not surprisingly, the broad roles of H3K79me2 and H3K36me3 make them increasingly important in treating developmental defects and diseases. Besides that, our previous studies (Zhang and Li, 2017, 2020; Zhang et al., 2018) demonstrate that 86.2% of expressed sequence tags are enriched in gene body regions. Of these tags in the gene body regions, 90.8% of tags are distributed in intron regions. These results indicate that the signal changes of H3K79me2 and H3K36me3 enriched in the gene body regions may induce the variations of chromatin accessibility and afford environments which provide greater flexibilities for gene expression regulation.

H3K27me3 is tightly associated with the repression of transcription in embryonic stem cells and neural, epidermal, and hematopoietic stem cells (Wang et al., 2009; Young et al., 2011; Gasiuniene et al., 2019; Dunican et al., 2020). It occurs together with H3K4me3 (activating mark) in regions referred to as bivalent domains, which often appear in the promoter regions of lineage-specific transcription factors (Azuara et al., 2006; Bernstein et al., 2006). Bivalent domains consist of these two HMs simultaneously, which can keep genes poised to respond to developmental cues (Igolkina et al., 2019; Zeng et al., 2019). Among the down-DEGs, we observe that H3K27me3 and H3K4me3 occur together and have marked increases from the −20th to the 50th bins. The down-regulation of down-DEGs indicates that leukemogenesis may prompt H3K27me3 to exert a stronger inhibitory effect than the activation of H3K4me3.

**FIGURE 3:**
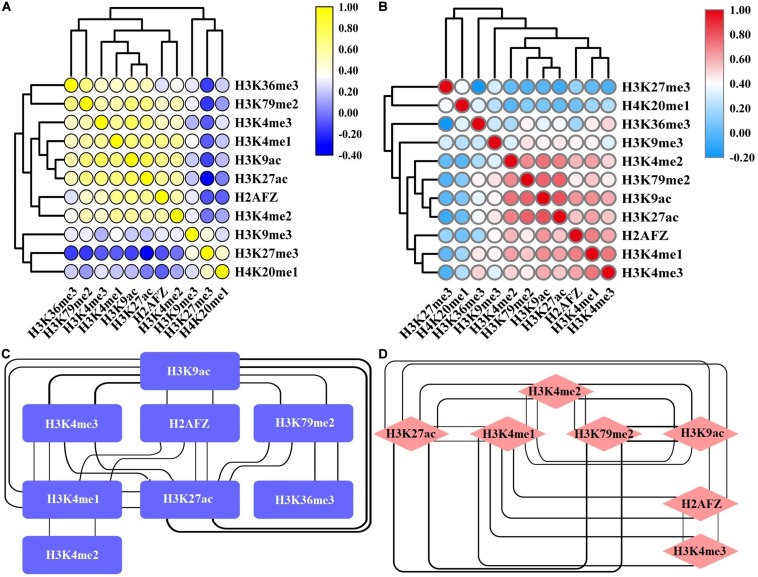
Correlation between various histone modifications (HMs) in the up-regulated and down-regulated differentially expressed genes (DEGs). Heat map of Spearman correlations for the signals of 11 HMs in the up-regulated DEGs **(A)** and down-regulated DEGs **(B)**. The interaction network among HMs whose Spearman correlations are greater than 0.6 in the up-regulated DEGs **(C)** and down-regulated DEGs **(D)**. The bolder the line, the stronger the correlation that it represents.

We also assess the impacts of combinations of HMs on gene expression changes and observe that several core HMs can effectively regulate gene expression. A possibility underlying these phenomena may be the functional similarities among these HMs. We thus carry out pairwise Spearman correlations for the signals of 11 HMs in the up- and down-DEGs. For the up-DEGs, there are three remarkable clusters that positively link to each other and promote gene expression (Figure 3A). They are (H3K36me3, H3K79me2), (H3K4me3, H3K4me1, H3K9ac, H3K27ac), and (H2AFZ, H3K4me2). For these three clusters, the pairwise Spearman correlations are greater than 0.6 (Figure 3C). Among the down-DEGs, two significant clusters that positively correlate to each other and activate gene expression are identified (Figure 3B). They are (H3K4me2, H3K79me2, H3K9ac, H3K27ac) and (H2AFZ, H3K4me1, H3K4me3). The pairwise Spearman correlations are also greater than 0.6 for these two clusters (Figure 3D). Overall, our research indicates that signal alterations of several core HMs are sufficient to regulate gene expression. Among these HMs, H3K79me2, and H3K36me3 exert their important regulatory roles in each of the 100 bins, and H3K27ac performs its regulatory roles from the −20th to the 10th bins.

## Data Availability Statement

The original contributions presented in the study are included in the article/Supplementary Material, further inquiries can be directed to the corresponding author/s.

## Author Contributions

L-QZ conceived the research and participated in data analysis, data interpretation, and manuscript preparation. HL and Q-ZL designed this idea and were involved in the discussion and revision of the entire article. G-LF, J-JL, and LL participated in data analysis, result discussion, and reviewed the manuscript. All authors contributed to the article and approved the submitted version.

## Conflict of Interest

The authors declare that the research was conducted in the absence of any commercial or financial relationships that could be construed as a potential conflict of interest.

## Abbreviations

CML: chronic myelogenous leukemia
HM: histone modification
RF: random forest algorithm
TSS: transcription start site
DEGs: differential expression genes
RPKM: reads per kilobase of exon model per million mapped reads
AUC: area under the receiver operating characteristic curve
IncMSE: percent increase in the mean squared error.
