# Supplementary material for: Identification of Key Histone Modifications and Their Regulatory Regions on Gene Expression Level Changes in Chronic Myelogenous Leukemia
Source: Front Cell Dev Biol. 2021 Jan 12;8:621578. doi: 10.3389/fcell.2020.621578 (PMC7835480; doi:10.3389/fcell.2020.621578)
Supplement: Supplementary file 1 [file Table_1.DOC]

Table S1. List of datasets used in this study. All data files can be downloaded from the stated sub-directories of the following URL: http://genome.ucsc.edu/ENCODE/downloads.html

| Transcription factor | Sub-directory | Dataset ID | Version |
| --- | --- | --- | --- |
| H3K27ac | wgEncodeBroadHistone | wgEncodeBroadHistoneGm12878H3k27ac | Hg19 |
| H3K27me3 | wgEncodeBroadHistone | wgEncodeBroadHistoneGm12878H3k27me3 | Hg19 |
| H3K36me3 | wgEncodeBroadHistone | wgEncodeBroadHistoneGm12878H3k36me3 | Hg19 |
| H3K4me1 | wgEncodeBroadHistone | wgEncodeBroadHistoneGm12878H3k4me1 | Hg19 |
| H3K4me2 | wgEncodeBroadHistone | wgEncodeBroadHistoneGm12878H3k4me2 | Hg19 |
| H3K4me3 | wgEncodeBroadHistone | wgEncodeBroadHistoneGm12878H3k4me3 | Hg19 |
| H3K79me2 | wgEncodeBroadHistone | wgEncodeBroadHistoneGm12878H3k79me2 | Hg19 |
| H3K9ac | wgEncodeBroadHistone | wgEncodeBroadHistoneGm12878H3k9ac | Hg19 |
| H3K9me3 | wgEncodeBroadHistone | wgEncodeBroadHistoneGm12878H3k9me3 | Hg19 |
| H4K20me1 | wgEncodeBroadHistone | wgEncodeBroadHistoneGm12878H4k20me1 | Hg19 |
| H2AFZ | wgEncodeBroadHistone | wgEncodeBroadHistoneGm12878H2AFZ | Hg19 |
| H3K27ac | wgEncodeBroadHistone | wgEncodeBroadHistoneK562H3k27ac | Hg19 |
| H3K27me3 | wgEncodeBroadHistone | wgEncodeBroadHistoneK562H3k27me3 | Hg19 |
| H3K36me3 | wgEncodeBroadHistone | wgEncodeBroadHistoneK562H3k36me3 | Hg19 |
| H3K4me1 | wgEncodeBroadHistone | wgEncodeBroadHistoneK562H3k4me1 | Hg19 |
| H3K4me2 | wgEncodeBroadHistone | wgEncodeBroadHistoneK562H3k4me2 | Hg19 |
| H3K4me3 | wgEncodeBroadHistone | wgEncodeBroadHistoneK562H3k4me3 | Hg19 |
| H3K79me2 | wgEncodeBroadHistone | wgEncodeBroadHistoneK562H3k79me2 | Hg19 |
| H3K9ac | wgEncodeBroadHistone | wgEncodeBroadHistoneK562H3k9ac | Hg19 |
| H3K9me3 | wgEncodeBroadHistone | wgEncodeBroadHistoneK562H3k9me3 | Hg19 |
| H4K20me1 | wgEncodeBroadHistone | wgEncodeBroadHistoneK562H4k20me1 | Hg19 |
| H2AFZ | wgEncodeBroadHistone | wgEncodeBroadHistoneK562H2AFZ | Hg19 |

TableS2. The prediction abilities of histone modifications

| **Histone Modification** | **AUC** | **Top five important bins** |
| --- | --- | --- |
| H3K79me2 | 0.90 | -13, -9, -7, -18, -8 |
| H3K36me3 | 0.89 | -37, -31, -41, -40, -42 |
| H3K27ac | 0.85 | -5, -6, -2, -3, -8 |
| H3K9ac | 0.83 | -9, -6, -7, -8, -3 |
| H3K4me3 | 0.81 | -8, -9, -10, -25, -20 |
| H3K4me2 | 0.80 | -20, -16, -14, -9, -31 |
| H3K4me1 | 0.78 | -49, -40, 10, -23, -45 |
| H3K9me3 | 0.73 | -36, -49, -44, -34, -26 |
| H2AFZ | 0.68 | 7, 3, 2, -8, -2 |
| H3K27me3 | 0.68 | -31, -22, 1, -8, -10 |
| H4K20me1 | 0.64 | -48, -30, -24, -41, -47 |


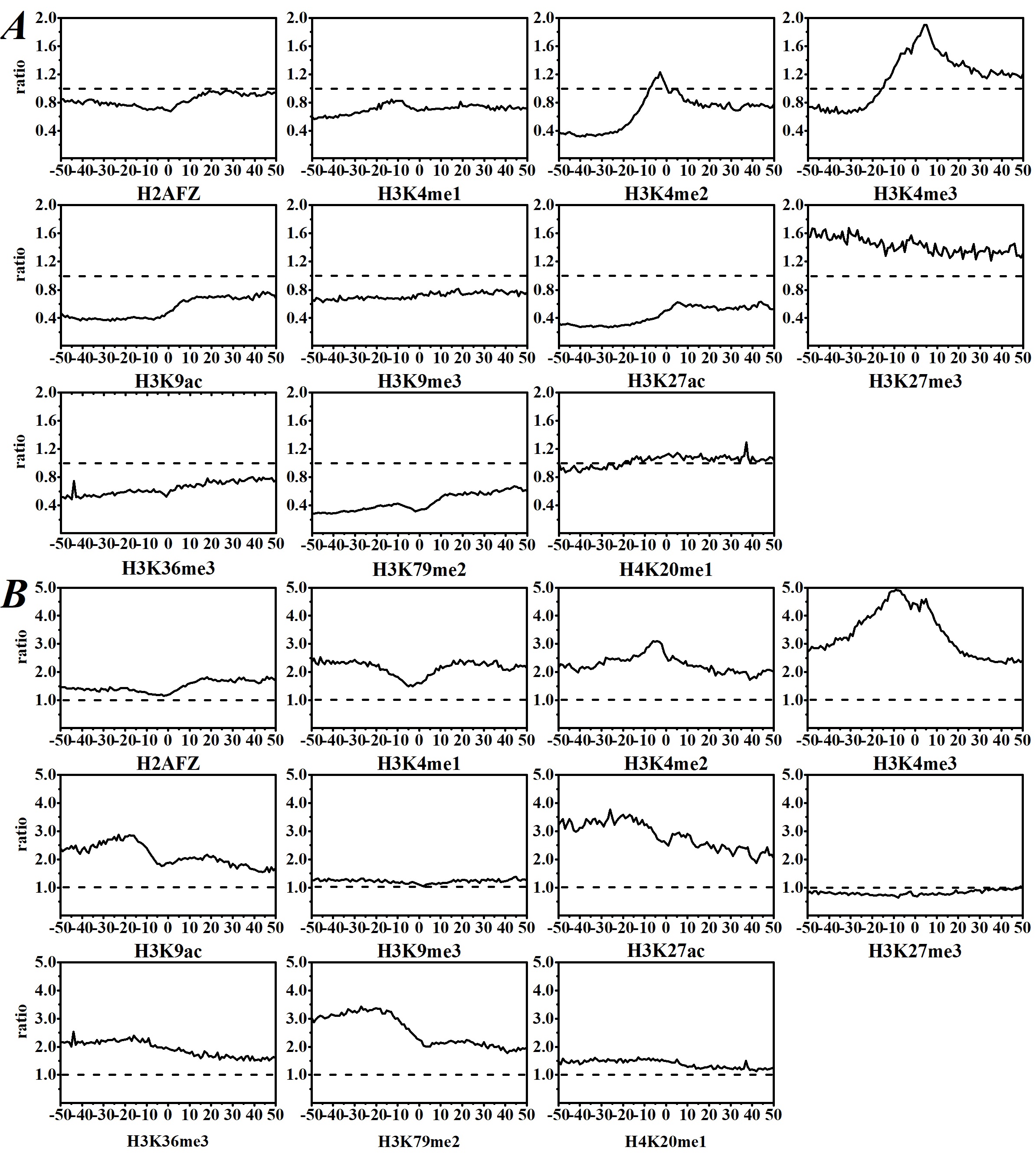


Figure S1. The ratio of HM signals in K562 to that in GM12878 for down-DEGs (A) and up-DEGs (B).
